# Impacted Permanent Teeth in the Mixed Dentition: Pediatric Diagnosis, Surgical Exposure, and Orthodontic Traction

**DOI:** 10.7759/cureus.117401

**Published:** 2026-10-03

**Authors:** Rama Almutlaq, Majid Alharthi, Faisal Albahkali, Abdullah Albadia, Wasan Koaban, Abdulaziz Alshalawi, Anas Alshammari, Sager Alyami, Mohammed Alsaadoun, Mohammed Alzahrani, Hamad Almuaiqly, Rahaf Mohammed, Azhar Aljalooud, Maryam Aljalooud, Fouad Althobaiti

**Affiliations:** 1 College of Dentistry, Princess Nourah Bint Abdulrahman University, Riyadh, SAU; 2 College of Dentistry, King Saud bin Abdulaziz University for Health Sciences, Riyadh, SAU; 3 College of Dentistry, Imam Abdulrahman Bin Faisal University, Dammam, SAU; 4 Faculty of Dentistry, King Abdulaziz University, Jeddah, SAU; 5 College of Dentistry, Umm Al-Qura University, Makkah, SAU; 6 Department of Dentistry, Ministry of Health, Tabuk, SAU; 7 Public Health, University of Essex, Colchester, GBR; 8 Pediatric Dentistry, Ministry of Health, Taif, SAU

**Keywords:** impacted permanent teeth, interceptive orthodontics, mixed dentition, orthodontic traction, pediatric dentistry, surgical exposure, tooth eruption

## Abstract

Permanent-tooth impaction during the mixed dentition is time-sensitive because eruption potential, root formation, arch space, and relationships with adjacent structures change during growth. This comprehensive narrative review evaluated evidence concerning pediatric diagnosis, interception, surgical exposure, orthodontic traction, treatment failure, and post-treatment outcomes. PubMed/MEDLINE, Embase, Scopus, and Web of Science were searched from inception through August 2026, with supplementary targeted searching. Diagnosis should integrate eruption symmetry and progression, space conditions, tooth-specific warning signs, and clinically directed imaging. Barrier removal and space provision may permit spontaneous eruption in favorable immature teeth, whereas primary-canine extraction may benefit selected palatal displacements but is not uniformly predictive. When definitive treatment is required, surgical access and orthodontic mechanics should be designed together. Evidence does not establish a universally superior exposure technique; tissue management, attachment placement, traction direction, and anchorage should follow anatomy and the intended eruption pathway. A defined path, usable space, stable anchorage, and staged movement away from vulnerable structures are more defensible than prescribing a named appliance or universal force threshold. Although alignment is often achievable in selected cases, periodontal, pulpal, root, esthetic, and stability outcomes remain relevant, together with repeat exposure, ankylosis, and adjacent-tooth injury. The literature is predominantly retrospective, concentrated on maxillary canines, and heterogeneous in definitions, protocols, and follow-up. The clinically responsible objective is not preservation of the impacted tooth at any cost, but selection of the strategy most likely to provide the growing child with a healthy periodontium, maintainable occlusion, and acceptable long-term restorative options.

## Introduction and background

The absence of a permanent tooth from the dental arch during the mixed dentition is a common clinical finding [1]. It may represent variation in normal development, delayed eruption, mechanical obstruction, ectopic displacement, ankylosis, or a primary disturbance of the eruption mechanism, and these conditions differ substantially in their expected response to observation and orthodontic force [1,2]. This distinction is consequential while root formation, eruption trajectory, arch space, and alveolar development are changing concurrently [1,3]. In a hospital-based series of 320 unerupted permanent incisors, 75.9% had at least one recorded complication, most commonly displacement or rotation of the unerupted tooth, loss of space, or deviation of the dental midline [3]. For palatally impacted maxillary canines, direct contact and a larger contact area with adjacent teeth have been associated with greater evidence of root resorption on cone-beam computed tomography [4]. The pediatric diagnostic task is therefore to determine whether a tooth is merely late, mechanically prevented from erupting, displaced into a hazardous relationship, or biologically unlikely to respond before the opportunity for conservative management narrows.

Diagnosis must provide information that changes management, rather than simply assign a labial, palatal, or vertical location. Eruption sequence and symmetry, persistence of the primary predecessor, palpable position, available space, previous trauma, root development, and the condition of neighboring teeth collectively determine the significance of a clinically absent tooth [1,3]. Imaging should then resolve the specific uncertainty left by the clinical assessment: the cause of obstruction, crown-root morphology, proximity to vulnerable roots, available alveolar pathway, and accessibility for a surgical attachment [4,5]. In a comparative investigation of impacted maxillary canines, interpretation of conventional two-dimensional records and cone-beam computed tomography produced differences in diagnostic assessment and proposed treatment [5]. The implication is that additional imaging is justified when the information obtained is likely to alter the decision to observe, intercept, expose, redirect, or abandon traction.

Management likewise extends along a continuum rather than beginning automatically with surgical exposure. Removal of a supernumerary tooth or another eruption barrier, combined where necessary with creation and preservation of space, may permit an impacted maxillary incisor to erupt without active traction; however, the supporting studies are predominantly retrospective and provide low- or very-low-certainty evidence [6]. Extraction of the primary canine may improve eruption of a palatally displaced successor in selected children, but the magnitude and predictability of that benefit remain uncertain [7]. Interception should consequently be regarded as a monitored therapeutic trial with a modifiable cause and predetermined criteria for continued observation or escalation. When those conditions are absent, or when displacement, adjacent-root injury, ankylosis, severe dilaceration, or failure of radiographic progression lowers the likelihood of spontaneous correction, definitive treatment should not be postponed merely to preserve the appearance of conservative care [2,4,8]. Timely escalation in this setting is an active form of risk control.

Once surgical exposure and orthodontic traction become necessary, the two procedures should be planned as components of one biologic and biomechanical strategy. Trials and systematic reviews have not demonstrated that retaining an open eruption window or replacing the flap over a palatally displaced maxillary canine produces consistently better clinical outcomes, and confidence in the available estimates remains limited [9]. Initial tooth position, vertical depth, angulation, age, and treatment protocol appear to influence difficulty and outcome, yet heterogeneity among studies has prevented reliable universal prediction rules [10]. Treatment success must therefore include more than entry of the crown into the arch. Systematic evidence indicates that successfully aligned impacted incisors and canines may retain modest periodontal differences, while treated maxillary incisors may exhibit altered root development or reduced alveolar support despite clinically acceptable alignment [11,12]. The literature is also anatomically uneven. Maxillary canines dominate comparative research, whereas evidence for impacted incisors is smaller and evidence for mandibular second molars is largely observational [10,12,13]. These limitations make direct translation of a single tooth-specific protocol to every mixed-dentition impaction inappropriate.

This comprehensive narrative review integrates the pediatric, surgical, and orthodontic evidence relevant to impacted permanent teeth identified during the mixed dentition. Its aim is to examine early recognition and diagnostic localization, determine when observation or interception remains defensible, clarify the principles governing tissue-preserving surgical exposure and controlled orthodontic traction, and evaluate the factors associated with prolonged treatment, complications, failure, and salvage. Periodontal, pulpal, root, esthetic, patient-reported, and stability outcomes are considered alongside successful eruption or alignment.

## Review

Methodological approach

The clinical pathway served as the organizing unit for this comprehensive narrative review. Evidence was assembled to address how a permanent tooth showing impaction, ectopic displacement, or failure of eruption should be recognized and managed during the mixed dentition while preserving adjacent teeth, alveolar and periodontal tissues, pulpal health, and future treatment options. The review was designed to examine time-sensitive problems requiring coordinated pediatric, orthodontic, and surgical decision-making.

The review was restricted primarily to permanent teeth that become unerupted, impacted, ectopically positioned, or mechanically obstructed during the mixed or transitional dentition. Evidence involving older adolescents or adults was considered when it addressed surgical or orthodontic techniques initiated during growth, provided long-term follow-up of teeth treated during childhood, or examined outcomes directly applicable to mixed-dentition management. Third-molar impaction and asymptomatic adult impactions were outside the scope of the review.

The review was organized around the domains that emerged as most relevant to interdisciplinary care: recognition of abnormal eruption, clinically directed imaging and three-dimensional localization, interceptive management, selection and execution of surgical exposure, periodontal-tissue preservation, orthodontic traction mechanics and anchorage, monitoring and criteria for escalation, recognition and management of treatment failure, salvage strategies, and post-treatment periodontal, pulpal, esthetic, and stability outcomes. Evidence limitations and priorities for future research were considered across these domains.

Information sources and literature search

Four bibliographic platforms were interrogated: MEDLINE through PubMed, Embase, Scopus, and Web of Science Core Collection. Coverage extended from the beginning of each database through August 2026. The initial strategy combined three principal concept groups: impacted or abnormally erupting permanent teeth; children, adolescents, and the mixed dentition; and diagnosis, imaging, interception, surgical exposure, orthodontic traction, complications, or treatment outcomes. The database-specific strategies are presented in Table 1.

**Table 1:** Database-specific electronic search strategies CBCT, cone-beam computed tomography.

Database	Search strategy
PubMed/MEDLINE	("Tooth, Impacted"[Mesh] OR "Tooth, Unerupted"[Mesh] OR "Tooth Eruption, Ectopic"[Mesh] OR "Tooth Ankylosis"[Mesh] OR impacted tooth[tiab] OR impacted teeth[tiab] OR unerupted tooth[tiab] OR unerupted teeth[tiab] OR delayed eruption[tiab] OR eruption disturbance*[tiab] OR eruption failure[tiab] OR failure of eruption[tiab] OR ectopic eruption[tiab] OR displaced canine*[tiab] OR impacted canine*[tiab] OR impacted incisor*[tiab] OR dilacerated incisor*[tiab] OR impacted premolar*[tiab] OR impacted molar*[tiab] OR infraocclusion[tiab]) AND ("Dentition, Mixed"[Mesh] OR "Child"[Mesh] OR "Adolescent"[Mesh] OR mixed dentition[tiab] OR developing dentition[tiab] OR child*[tiab] OR pediatric*[tiab] OR paediatric*[tiab] OR adolescen*[tiab] OR growing patient*[tiab]) AND ("Diagnostic Imaging"[Mesh] OR "Cone-Beam Computed Tomography"[Mesh] OR "Radiography, Panoramic"[Mesh] OR "Orthodontics, Interceptive"[Mesh] OR "Orthodontic Extrusion"[Mesh] OR "Tooth Movement Techniques"[Mesh] OR diagnos*[tiab] OR detect*[tiab] OR localiz*[tiab] OR radiograph*[tiab] OR CBCT[tiab] OR cone beam computed tomography[tiab] OR intercept*[tiab] OR spontaneous eruption[tiab] OR space creat*[tiab] OR space maint*[tiab] OR maxillary expansion[tiab] OR surgical exposure[tiab] OR open exposure[tiab] OR closed exposure[tiab] OR apically positioned flap[tiab] OR orthodontic traction[tiab] OR forced eruption[tiab] OR traction mechanic*[tiab] OR anchorage[tiab] OR treatment outcome*[tiab] OR complication*[tiab] OR treatment failure[tiab] OR ankylos*[tiab] OR root resorption[tiab] OR periodont*[tiab] OR pulp*[tiab] OR esthetic*[tiab] OR aesthetic*[tiab] OR stability[tiab] OR autotransplant*[tiab] OR decoronation[tiab])
Scopus	TITLE-ABS-KEY ("impacted tooth" OR "impacted teeth" OR "unerupted tooth" OR "unerupted teeth" OR "delayed eruption" OR "eruption disturbance" OR "eruption failure" OR "failure of eruption" OR "ectopic eruption" OR "displaced canine" OR "impacted canine" OR "impacted incisor" OR "dilacerated incisor" OR "impacted premolar" OR "impacted molar" OR infraocclusion) AND TITLE-ABS-KEY ("mixed dentition" OR "developing dentition" OR child* OR pediatric* OR paediatric* OR adolescen* OR "growing patient") AND TITLE-ABS-KEY (diagnos* OR detect* OR localiz* OR imaging OR radiograph* OR panoramic OR periapical OR occlusal OR parallax OR CBCT OR "cone beam computed tomography" OR intercept* OR "spontaneous eruption" OR "space creation" OR "space maintenance" OR "maxillary expansion" OR "surgical exposure" OR "open exposure" OR "closed exposure" OR "apically positioned flap" OR "orthodontic traction" OR "forced eruption" OR "traction mechanics" OR anchorage OR "treatment outcome" OR complication* OR "treatment failure" OR ankylos* OR "root resorption" OR periodont* OR pulp* OR esthetic* OR aesthetic* OR stability OR autotransplant* OR decoronation)
Web of Science Core Collection	TS=("impacted tooth" OR "impacted teeth" OR "unerupted tooth" OR "unerupted teeth" OR "delayed eruption" OR "eruption disturbance" OR "eruption failure" OR "failure of eruption" OR "ectopic eruption" OR "displaced canine" OR "impacted canine" OR "impacted incisor" OR "dilacerated incisor" OR "impacted premolar" OR "impacted molar" OR infraocclusion) AND TS=("mixed dentition" OR "developing dentition" OR child* OR pediatric* OR paediatric* OR adolescen* OR "growing patient") AND TS=(diagnos* OR detect* OR localiz* OR imaging OR radiograph* OR panoramic OR periapical OR occlusal OR parallax OR CBCT OR "cone beam computed tomography" OR intercept* OR "spontaneous eruption" OR "space creation" OR "space maintenance" OR "maxillary expansion" OR "surgical exposure" OR "open exposure" OR "closed exposure" OR "apically positioned flap" OR "orthodontic traction" OR "forced eruption" OR "traction mechanics" OR anchorage OR "treatment outcome" OR complication* OR "treatment failure" OR ankylos* OR "root resorption" OR periodont* OR pulp* OR esthetic* OR aesthetic* OR stability OR autotransplant* OR decoronation)
Embase	('tooth impaction'/exp OR 'tooth ankylosis'/exp OR 'impacted tooth':ti,ab,kw OR 'impacted teeth':ti,ab,kw OR 'unerupted tooth':ti,ab,kw OR 'unerupted teeth':ti,ab,kw OR 'delayed eruption':ti,ab,kw OR 'eruption disturbance':ti,ab,kw OR 'eruption failure':ti,ab,kw OR 'failure of eruption':ti,ab,kw OR 'ectopic eruption':ti,ab,kw OR 'displaced canine':ti,ab,kw OR 'impacted canine':ti,ab,kw OR 'impacted incisor':ti,ab,kw OR 'dilacerated incisor':ti,ab,kw OR 'impacted premolar':ti,ab,kw OR 'impacted molar':ti,ab,kw OR infraocclusion:ti,ab,kw) AND ('mixed dentition'/exp OR 'child'/exp OR 'adolescent'/exp OR 'mixed dentition':ti,ab,kw OR 'developing dentition':ti,ab,kw OR child*:ti,ab,kw OR pediatric*:ti,ab,kw OR paediatric*:ti,ab,kw OR adolescen*:ti,ab,kw OR 'growing patient':ti,ab,kw) AND ('diagnostic imaging'/exp OR 'cone beam computed tomography'/exp OR 'panoramic radiography'/exp OR 'orthodontics'/exp OR 'oral surgery'/exp OR diagnos*:ti,ab,kw OR detect*:ti,ab,kw OR localiz*:ti,ab,kw OR radiograph*:ti,ab,kw OR panoramic:ti,ab,kw OR periapical:ti,ab,kw OR occlusal:ti,ab,kw OR parallax:ti,ab,kw OR cbct:ti,ab,kw OR 'cone beam computed tomography':ti,ab,kw OR intercept*:ti,ab,kw OR 'spontaneous eruption':ti,ab,kw OR 'space creation':ti,ab,kw OR 'space maintenance':ti,ab,kw OR 'maxillary expansion':ti,ab,kw OR 'surgical exposure':ti,ab,kw OR 'open exposure':ti,ab,kw OR 'closed exposure':ti,ab,kw OR 'apically positioned flap':ti,ab,kw OR 'orthodontic traction':ti,ab,kw OR 'forced eruption':ti,ab,kw OR 'traction mechanics':ti,ab,kw OR anchorage:ti,ab,kw OR 'treatment outcome':ti,ab,kw OR complication*:ti,ab,kw OR 'treatment failure':ti,ab,kw OR ankylos*:ti,ab,kw OR 'root resorption':ti,ab,kw OR periodont*:ti,ab,kw OR pulp*:ti,ab,kw OR esthetic*:ti,ab,kw OR aesthetic*:ti,ab,kw OR stability:ti,ab,kw OR autotransplant*:ti,ab,kw OR decoronation:ti,ab,kw)

Scope of evidence

Sources were considered eligible when they addressed the recognition, diagnosis, localization, interception, surgical exposure, orthodontic traction, monitoring, complications, salvage, or post-treatment outcomes of impacted or abnormally erupting permanent teeth in children or adolescents. Eligible conditions included mechanical obstruction, ectopic eruption, tooth displacement, dilaceration, ankylosis, and primary failure of eruption when these conditions were relevant to differential diagnosis or the anticipated response to orthodontic traction. Eligible publications included randomized and nonrandomized clinical trials, prospective and retrospective cohort studies, comparative studies, diagnostic and imaging investigations, long-term follow-up studies, systematic reviews, and meta-analyses. Since uncommon impactions, severe dilacerations, ankylosis, transmigration, autotransplantation, and salvage procedures are rarely represented in controlled trials, clinically informative case series and selected case reports were considered when they described an otherwise underrepresented complication or management pathway. Technical or phantom-based imaging studies were considered when they directly informed localization accuracy, radiation optimization, or interpretation of structures relevant to treatment planning. Studies enrolling older adolescents or adults were retained selectively when they evaluated techniques or outcomes directly applicable to teeth whose impaction originates during the mixed dentition. These studies were relevant to comparisons of exposure techniques, traction mechanics, periodontal and pulpal outcomes, long-term stability, and survival after autotransplantation.

Studies were excluded if they focused exclusively on third-molar impaction, asymptomatic adult impactions without relevance to growing patients, primary teeth without implications for their permanent successors, or general oral-surgical and pathological outcomes unrelated to pediatric-orthodontic management. Studies addressing surgical extraction alone were excluded unless extraction formed part of a defined salvage or space-management strategy. Animal investigations, laboratory studies without direct diagnostic relevance, conference abstracts, and non-peer-reviewed sources were not included as evidentiary sources.

Framework for critical integration and synthesis

The synthesis was narrative and organized into thematic domains corresponding to the clinical pathway. Evidence was interpreted according to tooth type, location and severity of displacement, cause of eruption failure, root-development stage, dentition phase, availability of space, and relationship to adjacent teeth and cortical boundaries. Diagnostic evidence was evaluated in relation to the clinical question addressed, imaging modality, effect on treatment planning, and whether improved visualization was linked to a demonstrable change in patient management. Interceptive studies were compared according to the presumed eruption barrier, intervention performed, space management, duration of observation, spontaneous-eruption rate, and criteria for escalation. Surgical evidence was interpreted according to the labial or palatal position of the tooth, depth of impaction, flap design, preservation of keratinized tissue and follicular structures, attachment placement, and timing of orthodontic force. Orthodontic traction studies were synthesized in relation to force magnitude and direction, activation interval, anchorage design, movement sequence, treatment duration, and the need for repeat exposure or modification of mechanics.

Clinically meaningful outcomes included spontaneous eruption, successful alignment, treatment duration, repeat surgery, attachment failure, failure of movement, ankylosis, extraction or abandonment of traction, and damage to neighboring teeth. Post-treatment evaluation included periodontal health, marginal bone support, gingival recession, pulpal status, root development, root resorption, esthetic appearance, patient satisfaction, tooth survival, and positional stability. Successful entry of a tooth into the arch was therefore distinguished from broader biologic, esthetic, and patient-centered success. Particular attention was given to contradictions arising from differences in case selection, impaction anatomy, definitions of success and failure, surgical technique, orthodontic traction protocol, outcome measurement, and follow-up duration. Existing systematic reviews and meta-analyses were used to contextualize the direction and certainty of the evidence, but pooled estimates were interpreted in relation to their clinical and methodological heterogeneity. Because the review addressed multiple tooth types, interventions, and outcome domains, no new meta-analysis or formal quantitative risk-of-bias assessment was undertaken. Methodological weaknesses and evidence certainty were instead considered explicitly within each thematic domain and synthesized in the limitations section.

Recognizing abnormal eruption in the mixed dentition

Recognition of abnormal eruption in the mixed dentition should begin by distinguishing delayed emergence from true impaction. A clinically absent tooth may be developing normally but slowly, may have deviated from its eruption path, may be mechanically obstructed, or may be affected by a primary disturbance of the eruption mechanism. The terminology used has historically been inconsistent, particularly in separating delayed eruption, mechanical failure of eruption, and primary failure of eruption, which complicates both diagnosis and comparison between studies [1]. This distinction is clinically consequential because a tooth obstructed by a supernumerary tooth or odontoma may erupt after removal of the barrier, whereas a tooth affected by primary failure of eruption may not respond predictably to orthodontic force and may become ankylosed when incorporated into continuous mechanics [14]. Accordingly, the initial task is not merely to establish that a tooth is absent from the dental arch, but to determine whether its eruption sequence, symmetry, and clinical progression are compatible with normal development.

Chronological eruption tables provide a useful point of reference, but they are insufficient as stand-alone diagnostic thresholds because normal eruption timing varies with sex, population, dental maturity, local space conditions, and the developmental stage of the tooth. More informative warning signs include eruption of the contralateral tooth substantially earlier, reversal of the expected eruption sequence, failure to demonstrate progressive movement, and increasing asymmetry between dental quadrants. In a retrospective study of 15,987 children and adolescents attending a university dental hospital, Tan et al. defined an unerupted permanent incisor by clinically meaningful developmental discrepancies: the contralateral incisor having erupted more than six months previously, both maxillary central incisors remaining unerupted for more than one year after eruption of the mandibular incisors, or eruption of a maxillary lateral incisor before the adjacent central incisor [3]. These criteria are more useful clinically than a single age cut-off because they use the child’s own eruption pattern as an internal comparator.

Tan et al. identified 320 unerupted permanent incisors in 266 patients, corresponding to a prevalence of 2.0% within the hospital population; the mean age at diagnosis was 10.6 years, with a range from 7.3 to 13.8 years [3]. Maxillary central incisors accounted for 70.6% of the affected teeth, followed by maxillary lateral incisors at 22.5%, whereas mandibular incisors were comparatively uncommon [3]. Most affected patients had a single unerupted incisor, although approximately one-quarter had two involved teeth, indicating that both localized and more extensive developmental patterns occur [3]. In a separate retrospective investigation of 745 panoramic radiographs from children aged six to 10 years attending a Saudi tertiary hospital, delayed eruption of at least one permanent maxillary central incisor was recorded in 23 patients, giving a prevalence of 3.09% [15]. The difference between the two estimates should not be interpreted as evidence of a true geographic disparity because the studies used different age ranges, case definitions, sampling frameworks, and referral populations. More importantly, both were hospital-based retrospective studies and therefore cannot provide a robust estimate of prevalence in the general pediatric population.

Clinical recognition is often prompted not simply by absence of the permanent tooth, but by secondary changes in the developing occlusion. In the series reported by Tan et al., 75.9% of unerupted incisors were already associated with at least one complication, compared with 24.1% that had no recorded complication [3]. Displacement, ectopic position, or rotation of the unerupted tooth was documented in 46.6% of cases, space loss in 36.9%, and dental midline deviation in 27.5% [3]. Among maxillary central incisors specifically, displacement or rotation was observed in 49.1%, while space loss affected 41.2% [3]. These findings suggest that prolonged observation is not biologically neutral: even where the unerupted tooth itself remains asymptomatic, migration of adjacent teeth and progressive reduction of available space may make subsequent alignment more difficult. A retained primary incisor, especially when accompanied by asymmetry, loss of space, or eruption of the adjacent lateral incisor, should therefore be viewed as a diagnostic signal rather than as an isolated finding. In the Saudi hospital cohort, retained primary teeth were the most frequently recorded local finding, affecting 43.4% of children with delayed maxillary central incisor eruption [15].

A history of trauma to the primary dentition deserves particular attention when an incisor is absent or erupts in an abnormal position. Tan et al. found significant associations between previous primary-tooth trauma and dilaceration of the permanent successor, ectopic tooth-bud position, and ankylosis or impaction of the primary predecessor [3]. The corresponding probability values were less than 0.001 for dilaceration, 0.012 for ectopic tooth-bud position, and 0.039 for ankylosed or impacted primary teeth [3]. In a longitudinal assessment of permanent successors following trauma to primary teeth, de Amorim et al. found that enamel discoloration and hypoplasia were the most frequent sequelae among affected successors, while root and crown dilaceration occurred in 14.3% and 9.9%, respectively [16]. Avulsion and intrusive luxation of the primary predecessor were the injuries most frequently associated with dilaceration of the permanent tooth [16]. Consequently, an apparently uneventful trauma occurring several years earlier may still explain an abnormal eruption path, altered crown morphology, or unfavorable root configuration. The trauma history should therefore include injuries that occurred before the child or parent may have begun to regard the primary dentition as clinically important.

An unerupted incisor may also represent part of a broader developmental dental phenotype rather than a purely local event. Bartolo et al. compared 41 patients with unerupted incisors with 41 age- and sex-matched controls and found a significant association between unerupted incisors and other dental anomalies, including ectopic teeth, hyperdontia, and enamel hypoplasia [17]. Although the sample was small and included a wide age range, the association supports examination of the complete developing dentition when an abnormal incisor eruption pattern is identified. Clinically, this means that evaluation should not stop after locating the missing tooth; the clinician should also assess tooth number, morphology, contralateral development, eruption of other permanent teeth, and evidence of additional ectopic eruption. This broader examination is particularly important because an obstruction or developmental anomaly affecting one tooth may coexist with abnormalities elsewhere that materially alter treatment timing and anchorage planning.

The diagnostic meaning of clinical absence differs for maxillary canines because these teeth normally erupt later and follow a relatively long intraosseous path. In a longitudinal study of 505 Swedish schoolchildren observed from eight to 12 years of age, Ericson and Kurol found that 29% of 10-year-olds had bilaterally non-palpable maxillary canines, whereas the corresponding proportion at 11 years was only 5% [18]. A palpable buccal prominence in the apical region of the primary canine was associated with a favorable eruption position [18]. Non-palpability at 10 years should therefore not automatically be equated with impaction, especially when it is bilateral and dental development is otherwise symmetrical. Persistence of non-palpability, a marked difference between sides, an abnormal palatal prominence, prolonged retention of the primary canine, or displacement of the maxillary lateral incisor provides a stronger basis for further investigation than chronological age alone. This distinction prevents both delayed diagnosis of a genuinely displaced canine and indiscriminate imaging of children whose canine development remains within normal limits.

Posterior eruption abnormalities require a different pattern of recognition. An ectopically erupting permanent maxillary first molar may appear radiographically locked beneath the distal contour of the primary second molar, but this presentation does not invariably indicate irreversible impaction. In a retrospective study of 65 ectopically erupting maxillary first permanent molars, Dabbagh et al. found that 46 teeth, or 71%, self-corrected without active treatment [19]. Approximately one-third of the spontaneous corrections occurred after nine years of age, indicating that age alone is an imperfect basis for declaring the eruption disturbance irreversible [19]. Nevertheless, irreversible cases produced an average space loss of approximately 3 mm, and the magnitude of impaction was the most reliable predictor of failure to self-correct in multivariable analysis [19]. Greater resorption of the primary second molar, severe locking, and bilateral involvement were also associated with an irreversible outcome [19]. The clinically meaningful distinction is therefore not simply between normal and ectopic eruption, but between a mild, non-progressive disturbance that may resolve and a progressively locked molar that is damaging the primary predecessor or reducing arch length.

Infraocclusion of a primary molar is another finding that may reveal disruption of the local eruption environment. In a retrospective study of 3,132 children aged three to 15 years, infraocclusion of primary molars had a prevalence of 4.3%, occurred most frequently between six and nine years of age, and predominantly involved mandibular primary molars [20]. Infraocclusion does not by itself establish impaction of the permanent successor, but progressive submergence may be accompanied by tipping of adjacent teeth, loss of arch space, and altered eruption of the succedaneous premolar. Its recognition should therefore prompt assessment of the presence, position, and developmental stage of the successor rather than automatic removal of the primary tooth.

A less common but high-consequence presentation is the failure of several posterior teeth to erupt despite an apparently unobstructed eruption pathway. Primary failure of eruption typically produces a posterior open-bite pattern, often affects teeth distal to the first involved tooth, and may be mistaken initially for mechanical impaction [21]. A systematic review of reported primary failure of eruption cases found that approximately 85% of the 170 cases for which detailed family information was available had another affected family member, although this proportion is vulnerable to publication and ascertainment bias [2]. Mutations involving the parathyroid hormone receptor 1 gene have been identified in affected families and have been associated with failure of orthodontically assisted eruption [14]. Thus, multiple unerupted posterior teeth, a posterior open bite, absence of a convincing mechanical barrier, and a positive family history should move the clinician away from a simple impaction diagnosis and toward investigation of an eruption disorder. This distinction should be made before traction is attempted because unsuccessful orthodontic loading may compromise adjacent anchorage teeth and reduce later treatment options.

When these findings are considered together, abnormal eruption in the mixed dentition is best recognized as a deviation in a developing sequence rather than as absence of a tooth at a predetermined birthday. For incisors, the most informative signals are contralateral asymmetry, reversal of eruption order, retention of the primary predecessor, space loss, and midline deviation. For maxillary canines, persistence and asymmetry of non-palpability are more discriminating than non-palpability at a single young age. For first permanent molars, the severity and progression of locking, primary-molar resorption, and developing space loss help distinguish a self-correcting ectopic path from an irreversible disturbance. Finally, failure of several posterior teeth to erupt in the absence of a clear obstruction should raise suspicion of a primary eruption disorder rather than multiple independent impactions.

The strongest conclusion supported by the available evidence is that early recognition depends on serial clinical assessment integrated with dental development, eruption symmetry, and tooth-specific warning signs. What remains uncertain is the predictive accuracy of individual thresholds across different populations, because much of the evidence is derived from retrospective hospital cohorts, small observational samples, and non-uniform definitions of delayed eruption and impaction. A single abnormal sign should therefore trigger structured reassessment rather than an immediate definitive label; conversely, persistent asymmetry, progressive displacement, loss of space, or damage to neighboring teeth should not be managed by indefinite observation. Table 2 summarizes tooth-specific diagnostic priorities for four common eruption disturbances, outlining the key diagnostic questions, first-line imaging modality, and the clinical triggers that warrant escalation to cone-beam computed tomography (CBCT) or lateral cephalometry for each.

**Table 2:** Diagnostic priorities and first-line imaging for eruption disturbances requiring tooth-specific assessment CBCT, cone-beam computed tomography; 3D, three-dimensional. Created by the authors.

Eruption disturbance	Key diagnostic questions	First-line imaging	Escalation trigger and additional imaging
1. Unerupted maxillary incisor	Principal barrier: supernumerary tooth or odontoma?; sufficient space present?; root normal or dilacerated?	Periapical and occlusal radiographs.	Lateral cephalogram and/or limited-field CBCT if dilaceration cannot be characterized or crown-root angulation will determine traction feasibility or direction.
2. Ectopic maxillary canine	Vertical height, angulation, mesiodistal overlap, and root development; buccal or palatal position (parallax).	Panoramic radiograph and parallax localization.	Limited-field CBCT if there is substantial incisor overlap, suspected root resorption, unusual apical position, or unresolved access or traction direction.
3. Ectopic first permanent molar	Locked beneath the distal contour of the primary second molar?; magnitude of impaction and degree of primary-molar resorption.	Bitewing and panoramic radiographs.	Limited-field 3D imaging, rarely, if anatomy is atypical, additional pathology is suspected, or the relationship remains unresolved conventionally.
4. Displaced premolar	Predecessor status, developing root, eruption path, and available space.	Panoramic radiograph.	Periapical radiograph if local morphology remains unclear on panoramic imaging.

Interceptive management

Interceptive treatment seeks to restore a favorable eruption environment while the permanent tooth retains meaningful eruptive potential. Depending on the cause, this may involve removing a physical obstruction, eliminating an over-retained or diseased primary predecessor, creating and preserving arch space, correcting a relevant transverse deficiency, or unlocking an ectopically erupting first permanent molar [21,22]. Interception should not, however, be equated with passive delay. It is most defensible when three conditions are satisfied: the presumed cause of eruption failure is modifiable, the tooth has a plausible eruption pathway after intervention, and observation is governed by predetermined clinical and radiographic endpoints. Teeth with ankylosis, marked dilaceration, severe displacement, progressive pathology, or established injury to adjacent roots are correspondingly less suitable for prolonged interceptive observation [22,23].

Removal of eruption barriers affecting maxillary incisors

When a supernumerary tooth, odontoma, retained primary incisor, or other localized obstruction is demonstrably responsible for failed maxillary incisor eruption, removal of the barrier is usually the first causal intervention. The procedure should be planned around the three-dimensional relationship between the obstruction, the permanent incisor follicle, and the roots of adjacent teeth, because premature or poorly localized surgery may damage the developing incisor without necessarily improving its eruption pathway [21,22]. Removal of the obstruction does not guarantee spontaneous eruption. A 2023 systematic review included 15 studies and 1,058 participants, but 14 studies were retrospective and only one was prospective, illustrating the predominantly observational nature of the evidence [6]. The pooled eruption prevalence was 57.6% after removal of the supernumerary tooth alone, compared with 82.4% when removal was combined with space creation and 96.9% when combined with orthodontic traction [6]. These estimates should not be interpreted as direct comparative treatment effects because the contributing cohorts differed in case severity, adjunctive treatment, follow-up, and selection for immediate traction, and the certainty of the evidence was rated low to very low [6].

The higher eruption rates reported in some individual cohorts partly reflect more comprehensive protocols rather than removal of the obstruction alone. In one retrospective series of 66 delayed incisors in children aged six to 10.5 years, the supernumerary and retained primary incisors were removed, space was created when required, and 59.1% of patients also underwent primary canine extraction [24]. Spontaneous eruption occurred in 89.4% of teeth after a mean of 9.2 months, although the observed range extended from one to 27 months [24]. This result is clinically encouraging but cannot isolate the effect of supernumerary removal because space management and additional extractions were integral components of the protocol and no untreated comparison group was available. Incisor angulation was associated with delayed eruption in that cohort, whereas root maturity and vertical impaction were not significantly associated with whether eruption occurred [24]. In contrast, the subsequent meta-analysis found substantially more favorable eruption odds in incisors with incomplete root formation, although this estimate had a wide confidence interval and was derived from heterogeneous observational data [6]. Root development should therefore modify the estimated probability and acceptable observation period, but it should not function as an isolated treatment rule.

In a young child with an incompletely developed, reasonably oriented incisor and adequate space, careful removal of the obstruction without deliberate exposure of the permanent crown can avoid an unnecessary second intervention if spontaneous eruption follows. Conversely, immediate exposure and bonding may be more efficient when the incisor is mature, markedly angulated, positioned high in the alveolus, dilacerated, or unlikely to erupt without traction. The relative periodontal consequences of deliberate open or closed exposure must also be considered and are discussed in the following section.

Space creation and preservation as active treatment

The incisor meta-analysis found a pooled eruption prevalence of 82.4% when supernumerary removal was accompanied by space creation, compared with 57.6% after removal alone, although the difference remains vulnerable to confounding and between-study heterogeneity [6]. Planning must account for crown width, the path by which the tooth will emerge, the need to correct rotation or angulation, and the anchorage requirements if subsequent traction becomes necessary. Once obtained, space should be maintained until eruption is clinically secure. Otherwise, mesial drift, incisor migration, or premature loss of a primary tooth may recreate the original mechanical restriction.

A four-arm randomized trial provides preliminary evidence for a space-directed approach in younger children with potentially impacted maxillary canines. Seventy-six patients with 142 canines were followed after slow maxillary expansion, bilateral primary canine extraction, no intervention in the presence of crowding, or observation in a control group with adequate space [25]. At 18 months, canine sector improved significantly only in the expansion group, and over 18 to 60 months the need for major subsequent intervention was lower after expansion than after primary canine extraction or observation [25]. Nevertheless, the participants were approximately nine years old, buccal and palatal displacements could not yet be distinguished, baseline sector distributions were not completely balanced, and the diagnosis relied on panoramic measurements [25]. The findings support correction of demonstrable arch deficiency but do not justify routine expansion for every potentially displaced canine.

Interception of palatally displaced maxillary canines

The landmark prospective cohort by Ericson and Kurol followed 46 palatally displaced canines in 35 children aged 10 to 13 years who had no or only minor space loss [26]. After extraction of the primary canine, 36 of the 46 permanent canines (78%) showed normalization of their eruption pathway; 23 improved within six months and another 13 within 12 months, with no additional normalization first detected after 12 months [26]. The absence of an untreated control group means that this influential study could not distinguish treatment-induced correction from spontaneous positional improvement. More robust support came from a randomized trial of 67 children aged 10 to 13 years with unilateral or bilateral palatal displacement [27]. Spontaneous eruption occurred in 69% of the extraction group and 39% of the control group, with mean eruption times of 15.6 and 18.8 months, respectively [27]. Extraction also produced more favorable positional changes, but no significant difference in adjacent-root resorption was detected [27]. Interpretation is complicated by the combination of patient-level and split-mouth randomization and by protocol-driven intervention in initially untreated sites after 12 months when improvement was absent [23].

Accordingly, systematic assessments have reached superficially different but reconcilable conclusions. A 2021 Cochrane review judged the effect of primary canine extraction to be very uncertain: the relevant comparison produced a risk ratio of 2.87, but its 95% confidence interval ranged from 0.90 to 9.23 and the evidence was rated very low certainty [7]. An updated 2025 meta-analysis of five trials estimated that extraction increased the odds of permanent canine eruption by 3.6 times, but also identified baseline imbalances and other methodological weaknesses in some contributing studies [23]. The collective evidence therefore indicates a probable average benefit, while the outcome for an individual canine remains insufficiently predictable. The most defensible candidates are children in the mixed dentition, generally between approximately 10 and 13 years, with adequate or readily obtainable space and a permanent canine crown that has not migrated beyond the midline of the lateral incisor root [23]. Evidence of adjacent incisor resorption, severe mesial displacement, an almost horizontal orientation, advanced root maturity, or absence of radiographic improvement should lower the threshold for definitive orthodontic-surgical management rather than prolonged interception [23,28].

The evidence should not be generalized uncritically to labially displaced canines, in which insufficient space and arch-form constraints are often more prominent. Most trials specifically investigated palatal displacement or combined heterogeneous eruption disturbances, and therefore do not establish that primary canine extraction has the same effect across all displacement phenotypes [7,25]. A randomized trial of 32 children with 48 palatally displaced canines found clinical emergence in 64% of canines after double extraction and 78% after primary canine extraction alone, a nonsignificant difference [29]. Favorable final position was similarly not improved by double extraction, whereas initial canine angulation and available space were significant predictors of emergence [29]. The Cochrane analysis of three double-extraction trials likewise found no reliable eruption advantage over extraction of the primary canine alone at 18 months [7]. Routine removal of the primary first molar solely to improve canine eruption is therefore unsupported. It may still be justified by an independent sequencing, pathology, or space-management indication, but that decision should not be presented as an evidence-established enhancement of canine interception.

Ectopic eruption of first permanent molars

An ectopically erupting first permanent molar locked beneath the distal contour of the primary second molar requires a different interceptive logic. The immediate aims are to disengage and upright the permanent molar while preserving the primary second molar and arch length whenever possible. Observation is reasonable in mildly affected, recently diagnosed cases because spontaneous correction is common. In a cohort of 65 untreated ectopic maxillary first permanent molars, 71% self-corrected, and approximately one-third of the corrections occurred after nine years of age [19]. Conversely, 18 affected sites (28%) developed space loss averaging approximately 3 mm, and irreversible outcomes were associated with greater impaction, more extensive primary-molar resorption, and bilateral occurrence [19]. Thus, age alone should not dictate treatment: the severity and progression of locking are more clinically informative than a fixed chronological threshold.

Mild impactions may be unlocked with elastic or metal separators placed across the contact, whereas more severe locking generally requires controlled distal tipping with a brass ligature, spring-containing removable appliance, sectional fixed mechanics, slingshot-type appliance, or Halterman appliance [21]. The comparative evidence for these appliances is limited, and much of the literature consists of technical descriptions and small case series rather than controlled trials. Appliance selection should therefore depend on the depth of engagement, amount of distal movement required, accessibility for attachment placement, condition of the primary molar, available anchorage, and the child’s cooperation. Extraction of the primary second molar should not be the automatic means of releasing the permanent molar because it can convert an eruption disturbance into an arch-length problem. When extraction is unavoidable because of severe resorption, pulpal pathology, mobility, or non-restorability, immediate space control and a plan for permanent-molar uprighting are required. Before applying posterior orthodontic mechanics, ectopic mechanical locking must be distinguished from ankylosis and primary failure of eruption. In primary failure of eruption, teeth distal to the most mesial affected tooth may show a related eruption disturbance, and attempts at routine orthodontic movement frequently fail or produce secondary ankylosis [30]. A tooth that fails to respond to apparently adequate force should therefore prompt diagnostic reassessment rather than repeated force escalation.

Monitoring and criteria for escalation

Every interceptive intervention should be accompanied by a documented review schedule and a predefined alternative pathway. For unerupted incisors, clinical and radiographic reassessment at approximately six months is commonly recommended after removal of a supernumerary tooth [21]. If adequate space exists but no meaningful eruption is evident within six to 12 months, surgical exposure with or without bonding and orthodontic traction should be considered [21]. Selected younger incisors with favorable position and continuing radiographic progress may justify longer observation, because spontaneous eruption has occasionally occurred beyond 12 and even 18 months [22,24]. For a palatally displaced canine, positional improvement should normally be sought within the first six to 12 months after primary canine extraction. No newly normalized eruption pathways were identified beyond 12 months in the original Ericson and Kurol cohort, providing a pragmatic - although not absolute - endpoint for reconsidering treatment [26]. Earlier escalation is warranted if overlap with adjacent roots increases, resorption develops, space deteriorates, or the canine becomes less favorably oriented.

For an ectopic first permanent molar, the review interval should reflect the amount of locking and primary-molar resorption. Progressive resorption, increasing impaction, emerging space loss, pain, or primary-molar mobility shifts the balance from observation toward active disengagement [19]. The main clinical principle is that interception should reduce treatment burden without transferring risk into the future. A favorable young tooth may be given time to use its own eruptive potential, but observation remains appropriate only while the eruption trajectory is measurably improving and adjacent structures remain protected. When those conditions are no longer satisfied, timely surgical exposure and orthodontic traction become active risk-reduction measures rather than evidence of failed conservative care. The principal diagnostic questions, recommended imaging sequence, and clinical implications are summarized in Table 3.

**Table 3:** Diagnostic imaging sequence and evidence for three-dimensional localization of impacted permanent teeth in the mixed dentition CBCT, cone-beam computed tomography.

Diagnostic domain	Principal clinical question	Recommended approach	Supporting references
Clinically directed assessment	Does the presentation represent a true eruption disturbance, what is the probable mechanism, and which treatment decision remains uncertain?	Begin with dental history and clinical examination. Imaging should address a defined question concerning obstruction, tooth position, adjacent-structure risk, eruption prognosis, surgical access, or orthodontic traction.	[3,31]
Panoramic imaging	Is a broad overview of dental development, eruption sequence, tooth presence, space, displacement, or obstruction required?	Use panoramic imaging when the clinical question requires simultaneous assessment of developing teeth, eruption pathways, supernumerary teeth, odontomas, space conditions, and gross osseous abnormalities. It should not be prescribed automatically for every eruption delay.	[3]
Periapical and occlusal imaging	Is detailed assessment of root morphology, adjacent roots, an anterior obstruction, or local tooth orientation required?	Use periapical radiographs for localized crown–root morphology and adjacent-root assessment. Anterior occlusal views can supplement localization of unerupted incisors and anterior supernumerary teeth.	[3]
Parallax localization	Can the buccopalatal position be established adequately using conventional projections?	Obtain two appropriately angulated images and interpret the apparent movement of the unerupted tooth relative to a reference structure. Horizontal or vertical parallax may be sufficient when anatomy is uncomplicated.	[32]
Selective CBCT examination	Does unresolved three-dimensional anatomy have the potential to alter treatment or risk assessment?	Consider limited-field CBCT when conventional imaging cannot adequately define crown and root position, dilaceration, adjacent-root proximity or resorption, cortical boundaries, an obstructive lesion, surgical access, or the initial traction vector.	[5,31,33,34]
Diagnostic interpretation after CBCT	Does three-dimensional imaging materially change localization or recognition of adjacent-root injury?	Compare the CBCT findings with the unresolved questions identified after conventional imaging rather than interpreting additional anatomical detail as inherently important.	[5]
Effect on surgical and orthodontic planning	Does CBCT change surgical access, traction direction, space assessment, or the decision to retain the tooth?	Use three-dimensional findings to select an anatomically appropriate exposure route and traction vector while avoiding adjacent roots and cortical boundaries.	[33,35]
Adjacent-root resorption	Is an impacted tooth contacting or resorbing a neighboring root, and is the defect clinically consequential?	Use CBCT selectively when overlap or other conventional findings raise credible suspicion of resorption that cannot be characterized adequately in two dimensions.	[34,35]
Radiation and protocol optimization	Can the required information be obtained using a smaller field and lower exposure without compromising the diagnostic task?	Restrict the field of view to the region of interest and select pediatric exposure parameters according to the diagnostic objective, patient size, and ability to remain motionless.	[36,37]
CBCT interpretation and reporting	Which anatomical findings must be communicated to guide exposure and traction?	Review multiplanar sections and document crown and root position, root development and morphology, adjacent-root contact or resorption, physical obstruction, cortical relationships, associated pathology, and implications for surgical access and traction.	[31,38]
Overall evidence-based pathway	Does the anticipated diagnostic benefit justify escalation from two-dimensional to three-dimensional imaging?	Apply a sequential pathway: clinical examination → targeted conventional imaging → optimized limited-field CBCT only when a remaining three-dimensional uncertainty is likely to change treatment or risk assessment.	[31,35]

Surgical exposure and preservation of the periodontal tissues

Surgical exposure should be regarded as one component of a coordinated eruption protocol rather than as an isolated procedure. Its purpose is not merely to make the crown accessible, but to establish a biologically favorable route of eruption while preserving alveolar bone, keratinized tissue, gingival-margin architecture, and the roots of adjacent teeth [39]. The evidence informing surgical selection is uneven. Most comparative research concerns maxillary canines, whereas evidence for impacted incisors is derived from small retrospective cohorts and evidence for mandibular canines, premolars, and other permanent teeth in the mixed dentition is largely restricted to observational reports and clinical extrapolation [11]. Consequently, the apparent precision of many technique-selection algorithms exceeds the certainty of the supporting evidence.

Indication and interdisciplinary preoperative planning

Surgical exposure becomes appropriate when removal of an eruption barrier, space creation, or extraction of the primary predecessor has not produced sufficient eruptive progress and when the permanent tooth remains suitable for orthodontic alignment. Prognosis is influenced by tooth depth, orientation, root morphology, proximity to neighboring roots, and the presence of dilaceration or ankylosis; in a retrospective series of impacted maxillary incisors, five of six treatment failures involved dilacerated teeth [40].

Before surgery, the orthodontist and surgeon should agree on the intended surgical approach, the crown surface to be accessed, the attachment position, the route through which the ligature or chain will emerge, and the initial direction of tooth movement. This coordination is clinically important because a study of patients referred after unsuccessful treatment identified inadequate anchorage, inaccurate localization or inappropriate traction direction, and ankylosis as recurrent and sometimes overlapping causes of failure [41].

Adequate space and anchorage should either be present before exposure or incorporated into a clearly defined treatment sequence. In randomized trials of palatally displaced canines, orthodontic traction was initiated only after sufficient arch space and an appropriate working archwire had been established, even when fixed appliances were placed after the surgical procedure [42]. Surgery performed without a workable orthodontic plan may leave the attachment dormant, prolong plaque-retentive exposure, or necessitate a second procedure.

Selection of the exposure technique

The principal surgical approaches are excisional exposure, exposure with an apically positioned flap, open exposure, and closed exposure. In open exposure, the overlying tissues are removed or repositioned so that the crown remains accessible and can erupt spontaneously or under visible orthodontic guidance; in closed exposure, an attachment is bonded to the crown and the mucoperiosteal flap is replaced over the tooth before traction begins [43]. An apically positioned flap transfers keratinized tissue toward the anticipated cervical region of a labially positioned tooth, whereas excisional uncovering removes the tissue directly overlying a relatively superficial crown [39].

Technique selection should therefore be topographic rather than habitual. The relevant variables include whether the tooth is palatal, crestal, or buccal; its vertical relationship to the mucogingival junction; the amount and quality of keratinized tissue; the depth of osseous coverage; and the intended path into the alveolar ridge [39]. A technique that is suitable for a superficial palatal canine cannot automatically be transferred to a deeply positioned labial incisor or a canine lying apical to the mucogingival junction.

This anatomical approach also explains why the comparison between “open” and “closed” surgery can be misleading. Each category includes variations in flap design, bone removal, surgical packing, attachment placement, timing of traction, and allowance for autonomous eruption; these variations may influence periodontal and patient-reported outcomes independently of whether the flap is ultimately replaced.

Palatally displaced maxillary canines

The comparative evidence is strongest for palatally displaced canines, for which both open and closed procedures can be performed within keratinized palatal mucosa. A multicenter randomized trial of 62 unilateral cases found only a 0.1-mm difference in mean clinical attachment loss between open and closed exposure, which was neither statistically nor clinically significant [42]. Crown height, recession, and radiographic bone levels also did not differ significantly between the two surgical groups [42].

The same trial nevertheless demonstrated a small periodontal cost associated with treatment itself: surgically aligned canines showed approximately 0.5 mm more attachment loss than their untreated contralateral controls, and some degree of palatal root exposure was recorded in 20 of 62 treated canines compared with four contralateral canines [42]. This distinction is important because it suggests that impaction severity, surgery, orthodontic movement, or their combined effects may be more influential than the open-versus-closed distinction.

A subsequent multicenter trial randomized 120 patients aged nine to 16 years and successfully aligned all palatally displaced canines, with no clinically meaningful differences between open and closed exposure in periodontal status, root resorption, or total treatment duration [43]. The closed-exposure group reported greater discomfort mainly during active orthodontic traction, indicating that immediate postoperative experience and later traction-related experience should not be treated as the same outcome [43].

Systematic reviews have similarly found no convincing periodontal or aesthetic superiority of either technique, although the conclusions have been limited by small numbers of trials, heterogeneous protocols, and inconsistent outcome definitions [9,44]. Thus, for a palatal canine, the current evidence supports individualized selection according to depth, ease of access, anticipated eruption behavior, operator experience, and the planned orthodontic sequence rather than a universal preference for open or closed exposure.

Labially and buccally impacted canines

For labially impacted canines, mucogingival management assumes greater importance because the crown may lie beneath thin alveolar mucosa and outside the existing band of keratinized tissue. A radical excisional approach may provide rapid access but can create an emergence site with reduced keratinized tissue, inflammation, recession, or an unfavorable gingival margin. A 2016 systematic review found that excisional uncovering produced the least favorable soft-tissue results, whereas canines exposed with an apically positioned flap generally had periodontal findings comparable with untreated contralateral teeth; however, only three studies met the review criteria and none evaluated closed exposure adequately [45]. These findings therefore support avoidance of indiscriminate tissue excision but do not prove that an apically positioned flap is universally superior.

An older non-randomized comparison of 30 labially impacted anterior teeth found more apically positioned gingival margins, longer clinical crowns, visible scarring, and vertical relapse after apically positioned flaps than after closed exposure [46]. Conversely, the closed-exposure group demonstrated a narrower zone of attached gingiva at some sites and altered facial bone measurements, showing that flap replacement does not invariably reproduce normal periodontal anatomy [46]. Interpretation is constrained by the small, non-randomized sample, inclusion of different anterior tooth types, and relatively short minimum follow-up.

More recent evidence is cautiously reassuring. In a retrospective split-mouth study of 54 labially impacted canines treated by closed exposure, the treated teeth showed slightly less keratinized and attached gingiva, shorter roots, longer clinical crowns, and reduced interproximal bone support than their normally erupted antimere, but the investigators considered the overall recession clinically minor [47]. Greater pretreatment depth and mesial angulation were associated with less favorable alveolar crest measurements, suggesting that apparent differences between surgical techniques may partly reflect baseline impaction severity [47].

A 2024 meta-analysis of five studies found no significant difference in keratinized tissue width between treated buccally impacted canines and contralateral controls, although prospective studies showed a mean probing-depth increase of 0.16 mm around treated teeth [48]. The authors judged the observed periodontal differences - generally less than 1 mm - unlikely to be clinically important, but rated the certainty of evidence as low [48].

Taken together, the evidence does not establish a single preferred procedure for all buccal impactions. Excisional exposure should be limited to superficial teeth for which an adequate band of keratinized tissue will remain, an apically positioned flap is most defensible when keratinized tissue can be predictably transferred to the cervical region, and closed exposure is generally more appropriate for deeply positioned teeth or crowns lying apical to the mucogingival junction. These are anatomically coherent principles, but their comparative effectiveness remains incompletely tested.

Impacted maxillary incisors

Periodontal and aesthetic requirements are particularly exacting for maxillary central incisors because small discrepancies in gingival level, crown length, or contour are readily visible. The limited available evidence generally favors conservative closed exposure for deeply impacted or crestal incisors, although this preference is based predominantly on retrospective studies. In a cohort of 21 impacted central incisors treated with closed exposure, follow-up at a median of 4.5 years after retention showed generally favorable aesthetics, with only small increases in probing depth and an approximately 5%-6% reduction in radiographic bone support relative to the contralateral incisor [49]. Approximately one-third of treated incisors nevertheless displayed some gingival-contour irregularity, demonstrating that closed exposure reduces but does not eliminate the possibility of soft-tissue asymmetry [49].

By contrast, a small open-exposure cohort examined at a median of 10 years after retention showed reduced mesial bone support, longer clinical crowns, narrower attached gingiva, abnormal gingival contour in eight of 11 retained incisors, and positional relapse in five cases; one additional treated incisor had been lost before the final analysis [50]. The investigators appropriately cautioned that the sample was too small to provide a definitive estimate of risk [50].

A direct retrospective comparison of 11 open- and 11 closed-exposure cases found that open-exposed incisors had clinical crowns that were, on average, 1.37 mm longer and 7.5% less mesial bone support than closed-exposed incisors [51]. Although these differences support a closed approach in the aesthetic zone, the groups were small and could not fully account for differences in tooth position, trauma history, surgical execution, or orthodontic movement. A 2023 systematic review identified only three incisor studies, encompassing 54 patients, and rated all three at moderate risk of bias [11]. Closed exposure should therefore be viewed as a reasonable risk-reduction strategy for deeply impacted maxillary incisors, not as a guarantee of normal gingival and osseous anatomy.

Tissue economy, follicular preservation, and attachment placement

Regardless of the named technique, surgical tissue economy is central to periodontal preservation. Flap design should retain the available keratinized tissue and vascular supply, while osteotomy should be restricted to the amount required to identify the crown, create the planned eruption path, and bond an attachment without unnecessary injury to the cementoenamel junction or adjacent roots [39].

Preservation and repositioning of follicular tissue have been proposed as additional methods of supporting soft-tissue maturation. A small clinical series of 24 labially impacted canines reported acceptable periodontal and alveolar-bone findings after preservation and suturing of the dental follicle to the surrounding soft tissues [52]. Because the study lacked a concurrent alternative-surgery group, it supports biological plausibility rather than proving that follicular preservation improves outcomes.

Reliable attachment bonding is particularly important during closed exposure because bond failure may require repeat surgery. An observational study found that bonding at the initial exposure was more successful than delayed bonding, that eyelet attachments failed less frequently than conventional brackets, and that the palatal crown surface provided the least favorable bonding site [53]. Attachment selection and positioning should nevertheless be determined jointly with the intended traction vector, because the most accessible bonding surface is not necessarily the surface that permits the safest initial movement.

Postoperative recovery and surgical follow-up

Patient-reported recovery varies across studies and appears sensitive to the specific open-exposure protocol rather than merely to whether the flap is replaced. In a 2025 randomized trial of 100 patients, postoperative pain, discomfort, and recovery generally favored closed exposure, although analgesic consumption increased after suture removal in the closed group [54]. A 2026 randomized trial comparing glass-ionomer open exposure with closed exposure found greater pain with the open technique during the first postoperative week, but no significant overall difference across the complete postoperative period; surgical duration and complication rates were similar [55].

These findings indicate that recovery is time-dependent and that conclusions may change according to whether pain is measured immediately after surgery, after dressing or suture removal, or during subsequent orthodontic traction. Differences in surgical packing, glass-ionomer coverage, flap manipulation, and postoperative review schedules may explain some of the contradictions among trials.

Early follow-up should confirm uneventful flap healing, stability of the attachment or covering material, absence of infection, and the feasibility of plaque control before active traction is advanced. Once the tooth has entered the oral cavity, monitoring should expand from surgical healing to include gingival-margin position, keratinized tissue width, probing depth, inflammation, emerging crown orientation, and the periodontal condition of adjacent teeth. This is justified because pooled evidence suggests that successfully aligned impacted teeth may retain modest increases in probing depth, attachment loss, and bone loss even when the final result appears clinically acceptable [11].

Overall, the evidence supports anatomy-driven, tissue-preserving surgery rather than routine allegiance to an open or closed technique. For palatal canines, open and closed exposure produce broadly comparable periodontal outcomes; for labial canines and maxillary incisors, preservation of keratinized tissue and gingival architecture becomes more influential, but comparative evidence remains limited. The quality of flap design, bone conservation, attachment placement, and coordination with the intended eruption path may be at least as important as the formal name assigned to the surgical procedure.

Orthodontic traction: force delivery, direction of movement, and anchorage control

Orthodontic traction should be understood as the controlled three-dimensional relocation of an ectopic tooth rather than as simple vertical extrusion. The clinical objective is not merely to make the crown visible, but to move the tooth away from vulnerable structures, guide it through an appropriate alveolar and soft-tissue corridor, and establish a final crown-root position that can be maintained without periodontal compromise. The distinction is important because the most direct route to the dental arch is not necessarily the safest route for the adjacent roots, cortical plates, or gingival tissues.

Despite the large number of appliances described for impacted teeth, the comparative evidence supporting any particular traction system remains limited. Much of the biomechanical literature consists of laboratory measurements, finite element simulations, retrospective cohorts, and technique reports, whereas randomized clinical trials have principally compared surgical exposure methods rather than force systems [56-58]. Consequently, clinical decisions should be based on the predictability of the complete force system and the individual anatomy of the impaction rather than on claims that one named appliance is universally superior.

Prerequisites and timing of traction

Active traction should begin only after the orthodontist has confirmed the three-dimensional position of the tooth, identified the structures that must be avoided, provided an adequate destination within the arch, and established sufficient anchorage. Inadequate anchorage and incorrect localization or traction direction were identified in 48.6% and 40.5%, respectively, of a referral sample of previously failed impacted-canine treatments, although these figures describe causes within a selected failure population rather than their incidence among all treated patients [41]. The implication is that the traction plan should be formulated before surgical exposure and should specify the initial vector, the anchorage unit, the intended eruption corridor, and the criteria for changing direction.

Adequate space should represent a biologically and mechanically usable corridor rather than a measurement of crown width alone. The adjacent roots may remain convergent despite apparently sufficient coronal space, and traction into such a site can place the impacted crown in conflict with those roots. Space creation and root divergence may therefore need to precede active eruption, particularly when an impacted canine overlaps the lateral or central incisor or when a central incisor is positioned between adjacent developing roots.

The optimal interval between surgical exposure and force application has not been established independently of the exposure technique. Closed exposure generally incorporates an attachment that permits active traction after early surgical healing, whereas open palatal exposure may allow an initial period of spontaneous eruption before orthodontic alignment. In a multicentre randomized trial involving children aged nine to 16 years, open exposure followed by spontaneous eruption and closed exposure followed by active submucosal traction produced similar total treatment times, periodontal findings, and root-resorption outcomes; all included palatally displaced canines were eventually aligned [43]. The closed-exposure group reported greater pain and discomfort during the active traction phase, indicating that immediate mechanical engagement should not be assumed to provide a more efficient or more acceptable treatment experience [43].

In the mixed dentition, unnecessary traction should also be avoided when removal of an eruption barrier and preservation of space still offer a reasonable opportunity for spontaneous eruption. More than 65% of impacted maxillary incisors in a systematic review erupted after surgical removal of an associated obstacle, although the contributing studies were heterogeneous and predominantly retrospective [59]. Active traction is therefore most defensible when eruption has failed to progress during an appropriate observation period, the tooth’s position makes spontaneous correction unlikely, or delay would increase the risk to adjacent structures.

Force magnitude and activation control

The biologic rationale for using light, controlled force is persuasive, but a universally optimal force magnitude for impacted teeth has not been demonstrated in comparative clinical trials. Commonly cited values near 0.4-0.6 N should therefore be regarded as practical targets derived from biomechanical reasoning and selected clinical protocols rather than as validated thresholds applicable to every tooth and trajectory.

Laboratory testing of a helical cantilever designed for palatally impacted canines produced forces ranging approximately from 0.48 to 1.24 N depending on wire dimensions and configuration, demonstrating that relatively small alterations in appliance design can materially change the delivered force [60]. A subsequent retrospective cohort used a calibrated cantilever intended to maintain force below approximately 0.6 N and reported relatively efficient canine extrusion, but the absence of a concurrent comparison group prevents attribution of the outcome to force magnitude alone [61]. These studies support force measurement and appliance calibration, but they do not establish that 0.6 N is an absolute biologic boundary.

Conventional anchorage reinforcement with transpalatal arches and Nance appliances may reduce unwanted tooth movement, although neither provides absolute anchorage control [56]. Appliance selection should therefore consider not only the nominal eruptive force but also the accompanying buccolingual, mesiodistal, and rotational components. An apparently light pull may still produce an unfavorable moment or a substantial reciprocal load on the anchor teeth.

Cantilevers and segmented auxiliaries have the advantage that their force systems can be estimated more readily than those generated by complex continuous-arch configurations. Their predictability does not eliminate adverse effects, because every force applied to the impacted tooth creates an equal reciprocal force and moment on the anchorage unit. Force should consequently be measured when possible, reactivation should be modest, and the configuration should be reassessed as the tooth changes position rather than reproduced mechanically at every appointment.

Increasing force is not an appropriate first response to slow movement. Excessive force may enlarge areas of periodontal-ligament compression without correcting an obstructed or misdirected path. When movement is slower than expected, the clinician should first verify attachment integrity, force direction, available space, cortical interference, proximity to neighboring roots, and possible ankylosis.

Direction and sequencing of movement

Traction is frequently safer when divided into sequential phases. The initial phase should separate the impacted tooth from structures at risk; the intermediate phase should guide the crown into the intended eruption corridor; and the final phase should establish root position, torque, rotation, and occlusal integration. Attempting all these corrections simultaneously may produce an indeterminate force system and unnecessary loading of adjacent teeth.

For a palatally impacted canine overlapping the incisors, an initial vertical and distal movement may be more appropriate than immediate buccal traction toward the arch. In a finite element investigation using patient-derived models, vertical, distal, and buccal 1-N forces produced different stress distributions; vertical loading generated the lowest modeled root stresses, whereas buccal force increased cervical stress and the initial displacement was predominantly tipping rather than bodily movement [57]. A related finite element analysis demonstrated that the direction used to pull a palatally impacted canine also altered stress on the adjacent teeth [62]. These simulations support staged movement away from the incisors, but they cannot establish clinical superiority because their tissue properties, loading conditions, and boundary assumptions simplify the in vivo environment.

Direct physical contact and a larger contact area between an impacted canine and adjacent roots are associated with greater evidence of incisor root resorption on cone-beam computed tomography [4]. Where such proximity exists, the first traction vector should be selected to increase separation rather than to shorten the distance between the canine crown and the dental arch. A small comparative clinical study demonstrated the feasibility of using temporary anchorage devices to move palatally impacted canines away from neighboring roots before comprehensive bracket placement, although the study was insufficient to prove that this protocol prevents resorption [63].

Once a safe relationship with neighboring roots has been obtained, the vector may be progressively redirected toward the center of the alveolar process. Root uprighting and torque control are generally more manageable after the crown has emerged and a conventional bracket can be placed in a reproducible position. Premature attempts at full torque correction while the crown remains deeply embedded may generate large moments at the anchorage unit and may displace the root toward a cortical boundary.

The same principle applies to impacted incisors, although the required path is often determined by the orientation and morphology of the root. A comparative study found that a modified closed-eruption technique using palatal traction directed toward the alveolar crest produced outcomes comparable with spontaneous eruption for selected impacted maxillary central incisors [64]. The important element was not palatal traction as an isolated technique, but the creation of an eruption path that brought the crown toward the alveolar crest rather than directly through the labial plate or marginal gingiva.

Dilacerated incisors require particular caution because movement of the crown may displace the curved root or apex in an unintended direction. Finite element modeling of dilacerated impacted incisors confirms that initial displacement and stress distribution vary substantially with tooth angulation and force direction, but clinical validation of the modeled strategies remains necessary [65]. In a retrospective series of 64 impacted central incisors, the overall treatment success was 90%, but five of the six failures involved dilacerated teeth and greater impaction height prolonged treatment [40]. Dilaceration should therefore alter both the traction sequence and the prognosis communicated to the child and parents.

Anchorage design and appliance selection

Anchorage planning must control reciprocal effects in all three dimensions. Mesial movement or rotation of posterior teeth, changes in transverse arch width, occlusal canting, and unwanted movement of adjacent incisors may occur even when the impacted tooth appears to be moving successfully. Anchorage loss may also reduce the space originally prepared for eruption, creating a self-perpetuating conflict between the traction mechanics and the intended destination.

A continuous fixed appliance can distribute reciprocal forces across several teeth, but it may also transmit unwanted effects throughout the arch. Segmented mechanics permit clearer separation of the active and reactive units and can be especially useful when the initial objective is limited to vertical or distal disimpaction. The choice between these approaches should depend on the required force system, the teeth available for anchorage, the stage of dental development, and whether comprehensive alignment is already indicated.

Conventional reinforcement may include a rigid posterior segment, transpalatal arch, palatal acrylic button, or other cross-arch stabilization. A systematic review of three-dimensional anchorage during impacted-canine traction included 13 studies, most of which used conventional transpalatal anchorage and were judged to have low-to-moderate risk of bias [58]. The review found that transpalatal arches did not provide completely predictable sagittal, transverse, and vertical control, while the limited evidence concerning miniscrew anchorage appeared clinically promising but was insufficient for firm conclusions [58].

Temporary anchorage devices can provide direct traction without initially loading adjacent teeth, or they can reinforce a dental anchorage unit indirectly. This may be advantageous when incisor roots are already resorbed, when the canine must first be moved away from the dentition, or when comprehensive appliances would be premature. However, skeletal anchorage is not intrinsically risk-free or mechanically self-correcting; insertion site, bone availability, proximity to developing teeth, vector orientation, and miniscrew stability remain relevant, particularly in the mixed dentition.

Current evidence does not establish routine miniscrew use as superior to well-designed conventional anchorage for every impacted tooth. The more defensible conclusion is that temporary anchorage expands the available biomechanical options in anatomically demanding cases. Its use should be determined by whether it simplifies the required force system and reduces loading of vulnerable teeth, rather than by the assumption that skeletal anchorage automatically guarantees bodily or complication-free movement.

Position and traction considerations

For a deeply palatal canine, the first objective is commonly to reduce overlap with the incisors and move the crown into a position from which buccal and occlusal movement can occur without crossing their roots. For a superficial palatal canine, autonomous eruption after open exposure may reduce the amount of active traction required, although open and closed exposure have not shown consistent differences in final periodontal outcome [9]. The canine’s original depth and mesiodistal position appear more relevant to treatment difficulty than the label of the appliance used.

A buccally impacted canine presents a different relationship between traction and periodontal risk. The desired vector should bring the tooth through or toward an adequate band of keratinized tissue and into the alveolar housing rather than pull it rapidly through the alveolar mucosa. Across impacted maxillary incisors and canines, a systematic review found modest adverse periodontal differences after surgical-orthodontic alignment, but the certainty of evidence was low to very low and substantial clinical and methodological heterogeneity was present [11]. The periodontal corridor must therefore remain part of the traction plan even when the surgical exposure itself was appropriately selected.

For impacted maxillary central incisors, the esthetic importance of gingival-margin height and crown symmetry makes the final eruption path particularly consequential. The tooth should be guided toward the alveolar crest with sufficient control to avoid labial fenestration, excessive crown length, or an uncorrectable root prominence. Evidence regarding these techniques remains dominated by retrospective studies and small cohorts, and systematic reviews have emphasized the inadequacy of the literature for establishing a single preferred traction protocol [8,12].

Evidence concerning traction of impacted premolars, mandibular canines, and other permanent teeth in the mixed dentition is considerably less developed than that for maxillary canines and incisors. The general principles of space provision, measured force, staged movement, anchorage control, and periodontal-pathway preservation remain applicable, but tooth-specific protocols derived from canine studies should not be presented as directly validated for these less frequently studied impactions.

Monitoring movement and identifying mechanical failure

Monitoring should evaluate more than the amount of crown emergence. Each review should assess whether the tooth is moving along the intended path, whether the anchor teeth have displaced, whether prepared space is being maintained, and whether the soft tissues are healing around an appropriate eruption corridor. Adjacent tooth mobility, symptoms, pulpal findings, and radiographic evidence of progressive root resorption warrant particular attention when initial root contact or resorption was present.

Traction duration varies widely and should not be used alone to diagnose failure. In a cohort of surgically exposed maxillary canines, mean traction time was 13.9 ± 8.9 months, and the distance from the canine to the occlusal plane showed the strongest positive correlation with duration [66]. A subsequent single-centre predictive study associated longer traction with greater incisor overlap, wider canine angulation to the midline, absence of prior maxillary expansion, and missed appointments, but its model requires external validation before routine prognostic use [67]. Slow movement in a high impaction may therefore be biologically plausible, whereas complete absence of movement despite an apparently active force requires diagnostic reassessment.

The relationship between canine traction and adjacent-incisor root resorption remains less straightforward than is sometimes implied. A retrospective controlled study found that an impacted canine did not appear to increase orthodontically induced incisor resorption when the canine was first moved away from the incisor roots [68]. Another retrospective three-dimensional study reported no greater incisor resorption in canines classified as having high-complexity traction, although this finding may reflect careful anchorage and deliberate separation from neighboring roots rather than an absence of biologic risk [69]. A broader cohort comparison characterized maxillary canine impaction as only a weak predictor of orthodontically induced incisor resorption, but its panoramic measurements and observational design limit precision [70].

These findings suggest that root damage is not an inevitable consequence of traction and may be reduced by appropriate staging. They do not justify pulling an impacted tooth across an adjacent root or omitting surveillance in a high-risk case. The direction and control of movement may be more important than the mere fact that traction is performed.

When an impacted tooth fails to respond, force escalation should be deferred until the mechanics and diagnosis have been reconsidered. Attachment failure, loss of space, inadequate anchorage, incorrect vector, residual bony obstruction, cortical entrapment, severe root dilaceration, and ankylosis should be investigated systematically. The frequency with which incorrect anchorage and directional planning appeared in previously failed canine treatments reinforces the importance of reassessment rather than prolonged repetition of ineffective activation [41].

Biomechanical considerations

Several principles are sufficiently consistent to guide practice: traction should not begin without a defined three-dimensional pathway, usable space, and a stable anchorage unit; initial movement should prioritize separation from vulnerable roots and cortical boundaries; and the force system should be reassessed as the tooth changes position. It is also probable, although not conclusively proven by comparative trials, that measured light forces and staged movement are safer and more controllable than large, poorly characterized activations.

The evidence is less certain regarding the ideal numerical force, the superiority of a particular cantilever or spring, the optimal timing of activation after exposure, and the routine advantage of temporary skeletal anchorage. The literature is also disproportionately focused on maxillary canines, with much weaker evidence for incisors, premolars, and mandibular impactions. Orthodontic traction in the mixed dentition should therefore be individualized around anatomy and biologic risk, with the appliance serving the planned movement rather than determining it.

Treatment complications, failure recognition, and salvage strategies

Treatment failure should not be defined solely as the inability to bring an impacted tooth into the dental arch. A tooth may be successfully erupted yet leave clinically significant root resorption, periodontal breakdown, unfavorable gingival architecture, loss of vitality, anchorage damage, or an unstable crown-root position. Conversely, slow movement does not necessarily represent failure when the original impaction is deep, anatomically complex, or associated with root dilaceration. The central diagnostic task is therefore to distinguish a correctable technical problem from a biologic limitation or a treatment strategy whose risks have become greater than its likely benefit.

Defining and classifying failure

Reported failure rates vary because studies use different endpoints, including failure of surgical exposure, absence of orthodontic movement, need for repeat surgery, inability to complete alignment, and extraction after attempted traction. In a recent retrospective cohort of 214 surgically exposed maxillary canines, 23 teeth, representing 10.7%, failed to erupt after the initial surgical-orthodontic procedure and required re-intervention [71]. Orthodontic factors were considered predominant in 43.5% of these cases, surgical factors in 13.0%, and a combination of factors in the remainder, reinforcing that failure frequently arises from interaction between the two treatment phases rather than from an isolated error by one discipline [71]. These estimates derive from a specialist-centre retrospective cohort and should not be interpreted as a universal complication rate.

A useful clinical classification separates failures into four categories. Technical failures include attachment loss, force-system interruption, inadequate space, and anchorage loss. Positional or strategic failures arise when the original localization, direction of movement, or treatment objective is incorrect. Biologic failures include ankylosis, primary failure of eruption, severe dilaceration, progressive root resorption, or loss of periodontal or pulpal viability. Finally, patient- and treatment-process failures include prolonged non-attendance, poor appliance maintenance, inadequate oral hygiene, or inability to tolerate repeated surgery and extended orthodontic care.

The categories often overlap. In a selected referral series of 37 maxillary canines after unsuccessful treatment, inadequate anchorage was identified in 48.6%, incorrect localization or traction direction in 40.5%, and ankylosis in 32.4%, with more than one cause present in several cases [41]. Because this study examined patients referred specifically after failure, its percentages describe the distribution of problems within difficult failed cases and not their frequency among all treated impactions.

Attachment-related and early surgical complications

Loss of the bonded attachment is among the most immediately correctable causes of interrupted traction. Attachment failure may reflect inadequate moisture control, bonding to an unfavorable enamel surface, excessive loading, adhesive contamination, or mechanical fatigue of the chain or ligature. Its consequences extend beyond a temporary interruption because a deeply positioned tooth may require repeat surgical access and further removal or manipulation of the overlying tissues.

A clinical study of attachments bonded to impacted teeth found that bonding at the time of surgical exposure was more reliable than bonding at a subsequent procedure, and eyelet attachments had a lower failure rate than conventional brackets [53]. The palatal enamel surface produced the least favorable bonding performance, whereas pumice prophylaxis before etching did not improve attachment survival [53]. Although materials and adhesive protocols have since evolved, the study remains clinically relevant in showing that attachment design, bonding surface, and surgical-orthodontic coordination affect the likelihood of repeat intervention.

When the attachment is lost, the decision between immediate clinical rebonding and surgical re-exposure depends on whether sufficient enamel is accessible without damaging the soft-tissue corridor. Blind attempts to recover or redirect a submucosal chain can create unnecessary tissue trauma or apply force through an uncertain attachment orientation. Re-exposure is more defensible when the tooth remains deeply embedded, the original bonding surface is inaccessible, or the direction of the existing chain prevents the required movement. Repeat surgery was required for 62.9% of the canines undergoing corrective treatment in one referral series, most frequently to redirect or replace the traction connection [41]. This high proportion reflects a difficult, selected sample but demonstrates that unsuccessful traction cannot always be corrected by changing the orthodontic appliance alone.

Other reported surgical complications include infection, attachment detachment, soft-tissue penetration by the traction chain, wound-related pain, and premature soft-tissue closure over an open exposure. In the Cochrane review comparing open and closed exposure of palatally displaced canines, reported complications were uncommon but the available trials were too small and methodologically limited to provide reliable comparative estimates [9]. The review identified individual events including postoperative infection and chain-related soft-tissue complications, but the evidence was insufficient to establish that either exposure method carries a lower overall complication risk [9].

Pain should be considered separately from failure. A multicentre randomized trial found greater pain and discomfort during active traction after closed exposure, whereas open exposure produced greater immediate postoperative discomfort; total treatment time and final clinical outcomes were otherwise similar [43]. Persistent or increasing pain, swelling, suppuration, or unexplained bleeding should nevertheless prompt examination for infection, tissue ulceration, chain impingement, or inappropriate loading.

Failure of the tooth to move

An absence of clinically detectable movement should initiate a structured reassessment rather than automatic force escalation. Traction duration is highly variable: one study reported a mean canine traction period of 13.9 ± 8.9 months, with greater distance from the occlusal plane showing the strongest association with longer treatment [66]. Similarly, treatment of impacted central incisors averaged 21.6 ± 8.7 months in a retrospective series, and greater vertical impaction height significantly prolonged treatment [40]. A fixed number of months cannot therefore define failure without consideration of the initial position and the amount and direction of movement already achieved.

The first question is whether an effective force is reaching the tooth. Attachment integrity, chain continuity, spring activation, friction, and interference by the surrounding soft tissue should be checked. The second question is whether a sufficiently wide destination and an unobstructed movement path remain available. Space that was initially created may have been lost through anchorage displacement, eruption of adjacent teeth, or continued development of the dentition.

The third question concerns direction. A tooth may fail to emerge because it is being pulled against cortical bone, another root, the follicular boundary of a developing tooth, or an unfavorable undercut created by its own crown-root orientation. Three-dimensional reassessment is particularly important when the clinical direction of movement differs from the expected trajectory, adjacent teeth begin to move unexpectedly, or a deeply positioned tooth remains stationary despite verified force.

Failure of the impacted tooth to move while the anchorage unit displaces is a strong mechanical warning. Increasing activation in this situation is likely to amplify anchorage damage without overcoming the cause of resistance. The force should be discontinued until space, vector, tooth position, and ankylosis have been reassessed.

Ankylosis and primary failure of eruption

Ankylosis should be suspected when a correctly bonded tooth fails to move despite a verified force and a mechanically unobstructed path. Additional findings may include displacement of the anchorage teeth, loss of periodontal-ligament continuity on imaging, or a characteristic percussion response when enough crown is clinically accessible. None of these findings is independently definitive, particularly for a deeply impacted tooth, and ankylosis may only become apparent after a controlled trial of appropriately directed traction.

Ankylosis must also be differentiated from primary failure of eruption. Primary failure of eruption is characterized by incomplete eruption in the absence of a mechanical obstruction and most frequently affects posterior teeth, often producing unilateral or bilateral posterior open bite and involving teeth distal to the most mesial affected tooth [2]. Pathogenic variants in the parathyroid hormone receptor 1 gene have been identified in a proportion of affected families, but the phenotype is variable and a negative genetic result does not exclude the diagnosis [72,73].

The distinction is especially relevant in the mixed dentition, when a posterior permanent tooth may initially be labelled as impacted because it has not followed the expected eruption sequence. A clear eruption pathway, progressive posterior open bite, involvement of several teeth in the same quadrant, and a familial pattern should raise suspicion of a primary eruption disorder rather than an isolated mechanical impaction [72]. Orthodontic extrusion of teeth affected by primary failure of eruption is generally unsuccessful and may be followed by localized ankylosis and intrusion of the reactive anchorage teeth [74]. Surgical exposure and progressively heavier traction are therefore inappropriate when the underlying diagnosis is a failure of the eruption mechanism.

Isolated anterior impaction is less characteristic of primary failure of eruption and more commonly reflects obstruction, displacement, trauma-related dilaceration, or local ankylosis. Nevertheless, a single nonresponsive tooth should not be assumed to be mechanically obstructed merely because it has been surgically exposed. The diagnosis should be revisited whenever the clinical behavior is inconsistent with the original explanation.

Root, pulpal, and periodontal complications

Adjacent-root resorption may be present before traction begins, may progress while an ectopic tooth remains in contact with the root, or may occur as part of comprehensive orthodontic treatment. Because canine-associated incisor resorption can remain asymptomatic even when advanced, clinical absence of pain or mobility does not exclude significant structural loss [75]. Progressive resorption is a reason to interrupt traction and reassess whether the current vector is separating the teeth or maintaining damaging contact.

Recent longitudinal evidence provides some reassurance that carefully planned treatment does not necessarily accelerate pre-existing canine-induced resorption beyond the resorption associated with orthodontic treatment generally. In a longitudinal CBCT study, vertical resorption did not increase significantly more in incisors with pre-existing canine-induced resorption than in the non-resorbed contralateral incisors during subsequent orthodontic treatment [75]. However, some incisors already had substantial structural loss at baseline, and selection against treating the most severe cases may have influenced the results [75]. The finding supports cautious treatment after separation of the canine from the incisor, but it does not justify continued traction through direct root contact.

The impacted tooth itself may exhibit abnormal root development, surface resorption, pulpal obliteration, loss of vitality, or an unfavorable crown-root relationship after alignment. An older follow-up study of previously palatally impacted canines reported reduced attachment and crestal bone levels at selected sites, pulpal obliteration in six canines, pulp necrosis in one, and clinically noticeable positional relapse in approximately 40% of cases [76]. These figures should not be generalized to contemporary treatment because the study was retrospective and reflected older diagnostic, surgical, and orthodontic protocols.

For impacted maxillary central incisors, incomplete root development may continue after successful alignment, but the treated roots generally remain shorter than their contralateral counterparts [12]. The same systematic review found clinically acceptable periodontal measurements overall but reductions in alveolar bone thickness and support, with the evidence limited by moderate-to-high risk of bias [12]. A separate systematic review and meta-analysis concluded that surgically exposed and orthodontically aligned incisors and canines may have modest adverse periodontal differences, but confidence in the estimates was low to very low because of heterogeneity and study limitations [11].

A complication should therefore be judged by its clinical trajectory rather than by the presence of a small statistical difference alone. Mild transient inflammation after exposure or around an erupting attachment is different from progressive recession, increasing probing depth, persistent loss of keratinized tissue, or radiographic loss of supporting bone. The latter findings require modification of the traction path, periodontal intervention, or reconsideration of whether continued alignment remains appropriate.

Anchorage loss and damage to the surrounding dentition

Movement of the impacted tooth should not be assessed in isolation from the rest of the dentition. Loss of arch space, molar rotation or mesialization, intrusion of anchor teeth, incisor displacement, occlusal canting, and transverse distortion can convert technically successful eruption into a compromised overall result. A systematic review of anchorage during impacted-canine traction found persistent three-dimensional unpredictability with conventional transpalatal anchorage and insufficient comparative evidence to confirm the superiority of temporary skeletal anchorage [58].

Early anchorage loss may remain reversible through appliance redesign, restoration of space, segmentation of the arch, or temporary skeletal reinforcement. If it is ignored until the tooth approaches the arch, correction may substantially extend treatment and may require movements that place the newly erupted tooth or adjacent roots at additional risk. Anchorage preservation should therefore be treated as an outcome of traction, not merely as a prerequisite for it.

Deciding whether to continue, revise, or abandon traction

Continuation is reasonable when measurable movement is occurring along the intended pathway, adjacent structures remain stable, the periodontium is developing acceptably, and the projected treatment burden remains proportionate to the expected benefit. Revision is appropriate when failure can be linked to a correctable factor such as attachment loss, inadequate space, anchorage deficiency, or an incorrect initial vector. Abandonment should be considered when the tooth remains immobile after verified attachment, adequate space, appropriate force, and correction of the movement direction; when ankylosis or primary failure of eruption is strongly suspected; when progressive root, pulpal, or periodontal damage develops; or when the final position would remain functionally or esthetically unacceptable. No single radiographic index or duration threshold can determine this decision.

Anatomical severity materially affects the likelihood that revision will remain worthwhile. In a large retrospective study, canines positioned high above the adjacent root apices, with near-horizontal angulation, deep central placement within the alveolus, or a labiopalatal position were considered critically impacted and at greater risk of unsuccessful traction [77]. The overall findings were also strongly influenced by age and case selection, limiting direct prediction for an individual child [77].

For impacted central incisors, dilaceration and vertical height deserve particular weight. In a retrospective cohort, five of six failed incisors were dilacerated, while greater impaction height prolonged treatment even among successful cases [40]. Persisting with traction in a highly dilacerated tooth should therefore require evidence that the root can be accommodated within the alveolus without fenestration, severe apical displacement, or damage to neighboring structures.

The decision should incorporate the child’s treatment experience and the family’s tolerance for uncertainty. Repeated surgery, prolonged fixed appliances, missed schooling, discomfort, oral-hygiene challenges, and the possibility of eventual extraction are clinically relevant even when technically feasible salvage remains available.

Corrective traction and repeat exposure

When the cause of failure is mechanical, a structured reset may be preferable to incremental modification of an ineffective system. This may include recovering space, reinforcing the anchorage unit, obtaining updated localization, re-exposing and rebonding the tooth, rerouting the chain, or directing the initial movement away from adjacent roots before resuming alignment.

Corrective treatment successfully resolved 71.4% of the previously failed cases in one referral series, with a further mean treatment duration of 14.4 ± 7.2 months [41]. Corrective measures commonly included improved three-dimensional localization, altered traction direction, repeat surgery, and reinforcement with dental or skeletal anchorage [41]. These results demonstrate that initial failure does not necessarily make the tooth unsalvageable, but the small selected sample and substantial additional treatment burden should be acknowledged during consent.

Repeat exposure should be as conservative as the anatomy permits. Its purpose is to restore reliable access or correct the force direction, not to remove enough bone to make the tooth appear more accessible. Additional bone or follicular removal may compromise the periodontal attachment and does not correct ankylosis or an intrinsically unfavorable root position.

Management of ankylosed or otherwise non-tractable teeth

Surgical luxation followed by immediate orthodontic traction has been proposed for localized ankylosis, but its predictability is limited. In the failed-treatment referral series, only three of seven ankylosed canines treated by luxation and renewed traction were successfully positioned in the arch [41]. Published support for luxation is otherwise dominated by case reports and small clinical descriptions, and renewed ankylosis, root injury, loss of vitality, or failure of movement remains possible [78]. Luxation should consequently be presented as an uncertain salvage attempt rather than a routine response to nonmovement.

Autotransplantation may be considered when conventional traction is infeasible but the tooth can be removed atraumatically and placed in a surgically prepared recipient site. A systematic review and meta-analysis of autotransplanted teeth with incomplete root formation reported survival and success exceeding 95%, with low pooled rates of ankylosis, root resorption, and pulp necrosis [79]. These favorable estimates combined several donor-tooth types and predominantly observational studies, so they cannot be applied directly to every deeply impacted canine.

Long-term results specifically for autotransplanted maxillary canines are less favorable. A cohort with a mean survival time of 15.8 years reported an overall canine survival rate of 67.9%, illustrating the difference between short-term retention and durable long-term success [80]. Autotransplantation should therefore be reserved for carefully selected cases with suitable root morphology, an accessible donor tooth, an adequate recipient site, and a surgical team experienced in preserving periodontal-ligament viability.

When preservation of the impacted tooth is no longer reasonable, extraction must be integrated with a definitive orthodontic space strategy. Options include orthodontic space closure, substitution by another tooth, preservation of space for a future prosthesis, or autotransplantation of a different developing donor tooth [81]. In the mixed dentition, the decision should anticipate continued alveolar growth and changes in adjacent tooth position rather than address only the immediate space.

Decoronation has a narrow role when an ankylosed anterior tooth is already present in the arch or becomes progressively infraoccluded during growth. Clinical reports indicate that removal of the crown while retaining the ankylosed root may help preserve alveolar-ridge width and permit continued vertical bone development [82]. This approach is not a substitute for managing a deeply impacted ankylosed tooth and should not be extrapolated beyond its established use in growing patients with accessible, infraoccluded anterior teeth. Post-treatment periodontal, pulpal, esthetic, and stability outcomes are depicted in Table 4.

**Table 4:** Post-treatment outcomes following surgical exposure and orthodontic traction of impacted permanent teeth CBCT, cone-beam computed tomography

Outcome domain	Parameters assessed	Synthesis of reported findings	Clinical interpretation and certainty	Supporting references
Periodontal: plaque and inflammation	Plaque index, gingival index, bleeding on probing	Successfully aligned teeth generally demonstrate periodontal conditions approaching those of contralateral teeth, although a meta-analysis identified a small increase in plaque accumulation around previously impacted maxillary canines. These findings suggest modest site-specific susceptibility rather than generalized periodontal deterioration.	The average differences are probably of limited clinical importance in patients with effective plaque control. Confidence is low to very low because most studies were retrospective and evaluated successfully treated cases.	[11,83]
Periodontal: probing depth, attachment, and bone support	Probing pocket depth, clinical attachment level, radiographic crestal bone level	Compared with contralateral canines, formerly impacted canines showed small mean increases in probing depth, clinical attachment loss, and radiographic bone loss. The pooled differences were approximately 0.18 mm for probing depth, 0.40 mm for attachment loss, and 0.51 mm for bone loss.	The magnitude of the pooled changes is modest, but the consequences may be more relevant in teeth with thin periodontal tissues, limited alveolar housing, pre-existing inflammation, or extensive displacement. Certainty is low to very low.	[11,42]
Periodontal: recession and mucogingival appearance	Gingival recession, keratinized or attached gingiva, gingival-margin position, clinical crown height	Reduced keratinized tissue, gingival-margin asymmetry, and increased clinical crown height have been reported, particularly around labially positioned or inversely impacted incisors. A long-term study found reduced labial alveolar bone dimensions and more gingival recession around tractioned central incisors approximately 15 years after treatment.	Soft-tissue discrepancies may become more apparent with growth, maturation, and prolonged follow-up. The risk appears greater when the eruption path crosses thin labial tissues, but available evidence is low certainty.	[8,84,85]
Periodontal: influence of exposure technique	Open versus closed exposure, excisional exposure, apically positioned flap	Randomized and systematic-review evidence does not demonstrate clinically important periodontal superiority of open or closed exposure for palatally displaced canines. For labially impacted canines, excisional exposure has been associated with less favorable mucogingival outcomes, whereas an apically positioned flap may produce results closer to untreated teeth; however, the evidence is insufficient to establish a universally preferred technique. Retrospective incisor studies have reported longer clinical crowns and reduced periodontal support following open eruption compared with closed eruption.	Technique should be selected according to tooth depth, labial or palatal location, alveolar anatomy, and the availability of keratinized tissue. Evidence ranges from low to very low certainty and does not support one technique for all impactions.	[9,42,45,51]
Pulpal	Pulp sensibility, discoloration, pulp canal obliteration, pulp necrosis	Most aligned teeth retain clinically acceptable pulpal status, but delayed or absent sensibility responses, discoloration, and pulp canal obliteration have been documented. Pulp necrosis appears uncommon but has occurred in observational cohorts. Delayed sensibility and discoloration were reported more frequently after closed exposure in one retrospective canine study.	An abnormal sensibility response should not be interpreted as proof of necrosis without corroborating clinical and radiographic findings. The evidence is low certainty and does not establish whether pulpal alterations result from the original displacement, surgical manipulation, orthodontic force, or their combined effects.	[64,76,86,87]
Root development and morphology	Root length, apical maturation, root canal width, dilaceration	Immature impacted incisors usually continue root development after surgical–orthodontic treatment and may reach a developmental stage comparable with the contralateral incisor. Nevertheless, final root length frequently remains shorter, particularly in labially inverted or markedly dilacerated teeth.	Continued root formation supports timely management during the mixed dentition, but normalization of root morphology should not be assumed. Evidence is based mainly on small retrospective CBCT studies and is therefore low certainty.	[8,12,85,88]
Root resorption and adjacent teeth	Apical shortening, external root resorption, survival of adjacent incisors	Root shortening may affect the tractioned tooth or adjacent teeth, but separating damage caused by the original impaction from that associated with orthodontic movement is difficult. Pretreatment morphology and impaction relationships may influence post-treatment root length, whereas greater traction complexity has not consistently been associated with greater incisor resorption. Long-term observations suggest that many adjacent incisors with pre-existing canine-induced resorption can remain functional.	Baseline imaging must be considered when interpreting post-treatment changes. The evidence is heterogeneous and ranges from low to very low certainty.	[69,75,89,90]
Esthetic: tooth and gingival appearance	Crown height, gingival contour, color, torque, rotation, standardized esthetic indices	Successful alignment generally produces an acceptable appearance, although differences in gingival margin, clinical crown height, color, torque, or rotation may remain. Randomized evidence suggests that these discrepancies are often minor and may not be apparent to lay observers. Although one retrospective study favored closed canine exposure using the Maxillary Canine Aesthetic Index, this advantage has not been demonstrated consistently across study designs.	Esthetic success extends beyond bringing the tooth into the arch and should include gingival symmetry, crown orientation, and color. Confidence in technique-specific differences remains low.	[8,51,86,91]
Esthetic: patient-reported outcome	Satisfaction, self-perceived appearance, perceived treatment burden	Patient satisfaction is generally high despite professionally detectable imperfections. Patients may be less critical of minor asymmetry than orthodontists but frequently perceive treatment as prolonged.	Clinician-rated appearance cannot substitute for patient-reported outcome assessment. Available studies use heterogeneous and incompletely validated measures, resulting in low-certainty evidence.	[83,86,91]
Positional and biologic stability	Intrusion, rotation, transverse displacement, root and alveolar-bone stability	Long-term positional stability is poorly documented. One older cohort reported noticeable positional or appearance changes in approximately 40% of previously impacted canines, including intrusion, lingual displacement, mesial rotation, or discoloration. Conversely, small CBCT studies of treated impacted incisors and canines have reported relatively stable root and alveolar-bone conditions during follow-up.	A contemporary relapse rate cannot be estimated from the available evidence. Rotational correction, periodontal fiber orientation, root morphology, occlusal settling, and retention may all influence stability, but their independent effects remain uncertain. Evidence is very low certainty.	[76,88,90]

Limitations

The principal limitation of the literature is not a lack of clinical experience but a mismatch between numerous technical reports and relatively little comparative evidence capable of establishing treatment superiority. A systematic review of interventions for impacted maxillary canines identified 17 eligible studies but could not perform a quantitative synthesis because of small samples and substantial clinical heterogeneity; the certainty of evidence was rated very low for every evaluated outcome [10].

The limitations extend across the treatment pathway. For interceptive management, a Cochrane review included only four randomized studies involving 199 participants and judged the evidence concerning primary-tooth extraction and spontaneous canine eruption to be of very low certainty [7]. For surgical management, another Cochrane review included three studies involving 146 participants, two of which were at high risk of bias, and found insufficient evidence that either open or closed exposure was superior [9]. The literature concerning labially impacted canines is even less secure: a systematic review identified only three periodontal studies, none of which adequately evaluated the closed-eruption technique [45].

Evidence for impacted maxillary central incisors is similarly constrained. A systematic review of post-treatment incisor status included seven studies and 211 patients, of which five studies were retrospective and most carried moderate-to-high risks of bias [12]. A later review of labially inverted incisors found only six eligible studies with small samples and heterogeneous observational designs, despite apparently favourable treatment success among the selected cases [8]. Accordingly, the evidence more convincingly demonstrates that selected impacted teeth can be aligned than it establishes which intervention is optimal for a particular child.

Most available studies are retrospective and originate from specialist or university settings, where case selection, operator experience and referral patterns may differ substantially from routine pediatric practice [10]. A related concern is survivorship bias. Several periodontal investigations included only impacted teeth that had already been successfully exposed, tractioned and aligned, meaning that their findings describe periodontal status among treatment successes rather than periodontal risk across all attempted treatments [11]. Comparisons with the contralateral normally erupted tooth help control for patient-level characteristics but cannot separate the consequences of the original impaction from those of surgical exposure, orthodontic traction, prolonged appliance treatment and plaque accumulation. Consequently, a modest post-treatment difference cannot automatically be attributed to the surgical procedure or traction mechanics alone.

Treatment protocols are also insufficiently standardized. Studies have used different definitions of traction duration, sometimes measuring only forced eruption and sometimes the complete period of fixed-appliance treatment [10]. Orthodontic mechanics have ranged from elastomeric traction and auxiliary arches to cantilevers and skeletal anchorage, making treatment-time and complication comparisons difficult to interpret [10]. Surgical labels such as open and closed exposure similarly conceal differences in flap design, bone removal, follicular manipulation, attachment location, timing of force application and intended eruption pathway [92]. Apparent disagreement between studies may therefore reflect differences within the nominally same intervention rather than a true contradiction concerning its biologic effect.

The evidence is also anatomically unbalanced. Maxillary canines dominate the literature, whereas central-incisor evidence is smaller and frequently restricted to unilateral impactions or specific configurations such as labial inversion [8,12]. Evidence concerning mandibular canines remains heterogeneous and predominantly observational [93]. A systematic review of impacted mandibular second molars identified only eight studies, principally case series and cohort studies, with particularly limited evidence for surgical exposure [13].

Etiologic heterogeneity creates an additional problem. An incisor obstructed by a supernumerary tooth with an otherwise favourable eruption path is biologically different from a dilacerated, inverted or ankylosed incisor, even when both are categorized as impacted. Pooling these conditions can conceal clinically important differences in spontaneous eruption, traction difficulty, root development and failure risk.

Priorities for future research

Future investigations should distinguish tooth type, impaction location, etiology, root-development stage and dentition phase rather than treating all impacted permanent teeth as a single clinical entity. Baseline descriptions should include three-dimensional position where justified, space availability, eruption obstruction, follicular pathology, root morphology, proximity to adjacent teeth and periodontal phenotype.

Randomized trials remain appropriate where genuine clinical equipoise exists, such as comparisons of interceptive strategies, open and closed exposure, or immediate and delayed traction. The scarcity and uncertainty of existing trials indicate that adequately powered multicenter studies are required [7,9]. When randomization is impractical because anatomy determines treatment, prospective multicenter cohorts with predefined protocols, consecutive recruitment and adjustment for prognostic factors would provide more credible evidence than retrospective case series [10].

All attempted treatments should remain in the analysis, including failed traction, repeat exposure, ankylosis, extraction and conversion to an alternative strategy. Treatment duration should be separated into space creation, surgical-to-traction interval, active traction, final alignment and total appliance treatment. Surgical and orthodontic protocols should report flap design, amount of bone removal, attachment location, force magnitude and direction, activation interval, anchorage system and any change in mechanics.

A condition-specific core outcome set should be developed with pediatric patients, parents, pediatric dentists, orthodontists, periodontists and surgeons. The established orthodontic core outcome set already identifies self-perceived esthetics, alignment, stability, adherence, appliance breakage and adverse effects on teeth and supporting tissues as outcomes important to both clinicians and patients [94]. For impacted teeth, this framework should be expanded to include eruption without surgery, successful alignment, re-intervention, pulpal vitality, root development, adjacent-root resorption, periodontal health, treatment burden and eventual tooth retention.

Follow-up should extend beyond initial eruption and appliance removal because immediate alignment does not establish long-term periodontal, pulpal or positional stability. Outcome assessment at standardized short- and long-term intervals would allow transient postoperative changes to be distinguished from persistent tissue effects. Blinded periodontal and radiographic assessment, reporting of confidence intervals and prespecified thresholds of clinical importance would further improve interpretability. Finally, multicenter registries could be especially valuable for uncommon presentations such as mandibular transmigration, impacted premolars, multiple impactions and severe dilaceration, for which conventional trials may never recruit adequate samples. Predictive models should be developed from such representative datasets and validated externally before being used to recommend observation, interception, traction or extraction.

Overall, absence of evidence demonstrating superiority should not be interpreted as proof that competing techniques are equivalent. Until stronger comparative data become available, treatment should remain individualized according to eruption biology, three-dimensional anatomy, root maturity, periodontal considerations and the child’s tolerance for treatment burden.

## Conclusions

Impacted permanent teeth in the mixed dentition represent a developmental and time-sensitive problem. Effective management begins with pediatric detection of abnormal eruption and identification of its cause, followed by orthodontic assessment of eruption potential, space availability, anchorage requirements, and a biologically appropriate path of movement. When removal of an obstruction and space provision are unlikely to permit spontaneous eruption, surgical exposure should be planned in direct coordination with the orthodontic traction strategy. The choice of surgical exposure technique should reflect the three-dimensional tooth position, depth of impaction, periodontal anatomy, and intended force vector, with maximum preservation of bone and keratinized tissue. A successful outcome should preserve pulpal vitality, root development, periodontal support, gingival esthetics, adjacent teeth, occlusal function, and long-term stability.
